# Toward accurate cerebral blood flow estimation in mice after accounting for anesthesia

**DOI:** 10.3389/fphys.2023.1169622

**Published:** 2023-04-12

**Authors:** Zhiliang Wei, Yuguo Li, Adnan Bibic, Wenzhen Duan, Jiadi Xu, Hanzhang Lu

**Affiliations:** ^1^ Russell H. Morgan Department of Radiology and Radiological Science, Johns Hopkins University School of Medicine, Baltimore, MD, United States; ^2^ F. M. Kirby Research Center for Functional Brain Imaging, Kennedy Krieger Research Institute, Baltimore, MD, United States; ^3^ Division of Neurobiology, Department of Psychiatry and Behavioral Sciences, Johns Hopkins University School of Medicine, Baltimore, MD, United States; ^4^ The Solomon H. Snyder Department of Neuroscience, Johns Hopkins University School of Medicine, Baltimore, MD, United States; ^5^ Department of Biomedical Engineering, Johns Hopkins University School of Medicine, Baltimore, MD, United States

**Keywords:** phase contrast (PC), cerebral blood flow (CBF), respiration rate (RR), dose, isoflurane, mouse, MRI, heart rate (HR)

## Abstract

**Purpose:** To improve the accuracy of cerebral blood flow (CBF) measurement in mice by accounting for the anesthesia effects.

**Methods:** The dependence of CBF on anesthesia dose and time was investigated by simultaneously measuring respiration rate (RR) and heart rate (HR) under four different anesthetic regimens. Quantitative CBF was measured by a phase-contrast (PC) MRI technique. RR was evaluated with a mouse monitoring system (MouseOX) while HR was determined using an ultrashort-TE MRI sequence. CBF, RR, and HR were recorded dynamically with a temporal resolution of 1 min in a total of 19 mice. Linear regression models were used to investigate the relationships among CBF, anesthesia dose, RR, and HR.

**Results:** CBF, RR, and HR all showed a significant dependence on anesthesia dose (*p* < 0.0001). However, the dose in itself was insufficient to account for the variations in physiological parameters, in that they showed a time-dependent change even for a constant dose. RR and HR together can explain 52.6% of the variations in CBF measurements, which is greater than the amount of variance explained by anesthesia dose (32.4%). Based on the multi-parametric regression results, a model was proposed to correct the anesthesia effects in mouse CBF measurements, specifically ${CBF}_{corrected}=CBF+0.58RR-0.41HR-32.66Dose$ . We also reported awake-state CBF in mice to be 142.0 ± 8.8 mL/100 g/min, which is consistent with the model-predicted value.

**Conclusion:** The accuracy of CBF measurement in mice can be improved by using a correction model that accounts for respiration rate, heart rate, and anesthesia dose.

## 1 Introduction

Cerebral blood flow (CBF), which denotes the amount of blood supplying the brain tissue per unit of time, is a fundamental parameter of brain physiology (Lassen, 1959; Lotz et al., 2002). As a quantitative marker, CBF directly evaluates the delivery capacity of the cerebral vascular system and indirectly reflects the brain metabolism through the flow-metabolism coupling (Peterson et al., 2011). Therefore, the measurement of cerebral blood flow (CBF) in humans provides a powerful biomarker for normal aging and various brain diseases, e.g., Alzheimer’s disease, Parkinson’s disease, cancer, and ischemic stroke (Kawabata et al., 1991; Copen et al., 2011; Lu et al., 2011; Yeom et al., 2014; Kisler et al., 2017). Furthermore, the evaluation of the efficacy of agents or drugs should be conducted in the context of CBF due to the simple fact that agents or drugs are delivered by blood flow. CBF may also be used as an index for toxicity in that it will inform us about the extent to which an agent perturbs the physiology (Mathew and Wilson, 1991).

Rodent models provide valuable opportunities for the mechanistic understanding of pathological processes, trials of novel therapies, or validation of biomarkers that were originally proposed based on human studies (Ericsson et al., 2013; Liu et al., 2021; Wei et al., 2023). Paralleling the popularity of CBF measurement in human studies, there has been a continuous interest in developing techniques for measuring CBF in small animals (e.g., mice) (De Visscher et al., 2006; Hedna et al., 2015; Hirschler et al., 2018; Wei et al., 2019). One potential confounding factor in such studies is anesthesia, which is used in virtually all studies of mice. Most anesthetic agents are known to have a vasoactive effect (Fukuda et al., 2013; Li et al., 2013; Slupe and Kirsch, 2018), either dilating or constricting blood vessels. For example, as the most broadly used inhalational anesthesia due to its advantages of easy implementation and safety (i.e., animal will recover soon after stopping exposure), isoflurane is associated with a dose-dependent vasodilative effect (Li et al., 2013). In contrast, dexmedetomidine, which is typically administered with injection, has an opposite effect of reducing CBF (Fukuda et al., 2013). Apart from the vasoactive differences across anesthetic agents, even if the same agent is used, there may be a time-dependent change in the actual anesthetic dose throughout an experimental session (Wei et al., 2022). Therefore, in order to correctly interpret CBF measurements in mice, the effects of anesthesia must be elucidated and, ideally, accounted for.

Here, we aimed to systematically understand the relationship between CBF and related physiological factors, not only the anesthesia dose but also respiration rate (RR), heart rate (HR), and exposure time to anesthesia. We then applied these relationships to develop a correction scheme for anesthesia-independent CBF estimation.

## 2 Methods

### 2.1 MRI experiments

The institutional animal care and use committee approved the experimental protocol. MRI experiments were performed on an 11.7T Bruker Biospec system (Bruker, Ettlingen, Germany) with a horizontal bore equipped with an actively shielded pulse field gradient (maximum intensity of 0.74 T/m). Images were acquired using a 72-mm quadrature volume resonator as a transmitter, and a four-element (2 × 2) phased-array coil as a receiver. The homogeneity of B_0_ field over the mouse brain was optimized with a global shimming (up to 2^nd^ order) based on a subject-specific pre-acquired field map.

A total number of 19 C57BL/6 mice (8 female and 11 male mice; age: 32.5 ± 2.5 weeks [mean ± standard error]; body weight: 29.7 ± 1.4 g) was used in the present study. All mice had free access to food/water and were housed in a quiet environment with a 12-h day/night cycle. Anesthesia was administered with 1.5% isoflurane for 15 min as induction and 1.0% isoflurane as maintenance before the planned anesthetic regimens (detailed later) started. Approximately 10th minute after the anesthetic induction, the mice were relocated to a water-heated animal bed with temperature control and positioned with a bite bar, ear pins and a customized holder (generated with Ultimaker 2 Extended + 3D printer, Ultimaker, Utrecht, Netherland) to minimize motions. Soft tapes were used at the dorsal sides of the mice with gauze in-between to avoid direct contact between tapes and dorsal fur.

**FIGURE 1:**
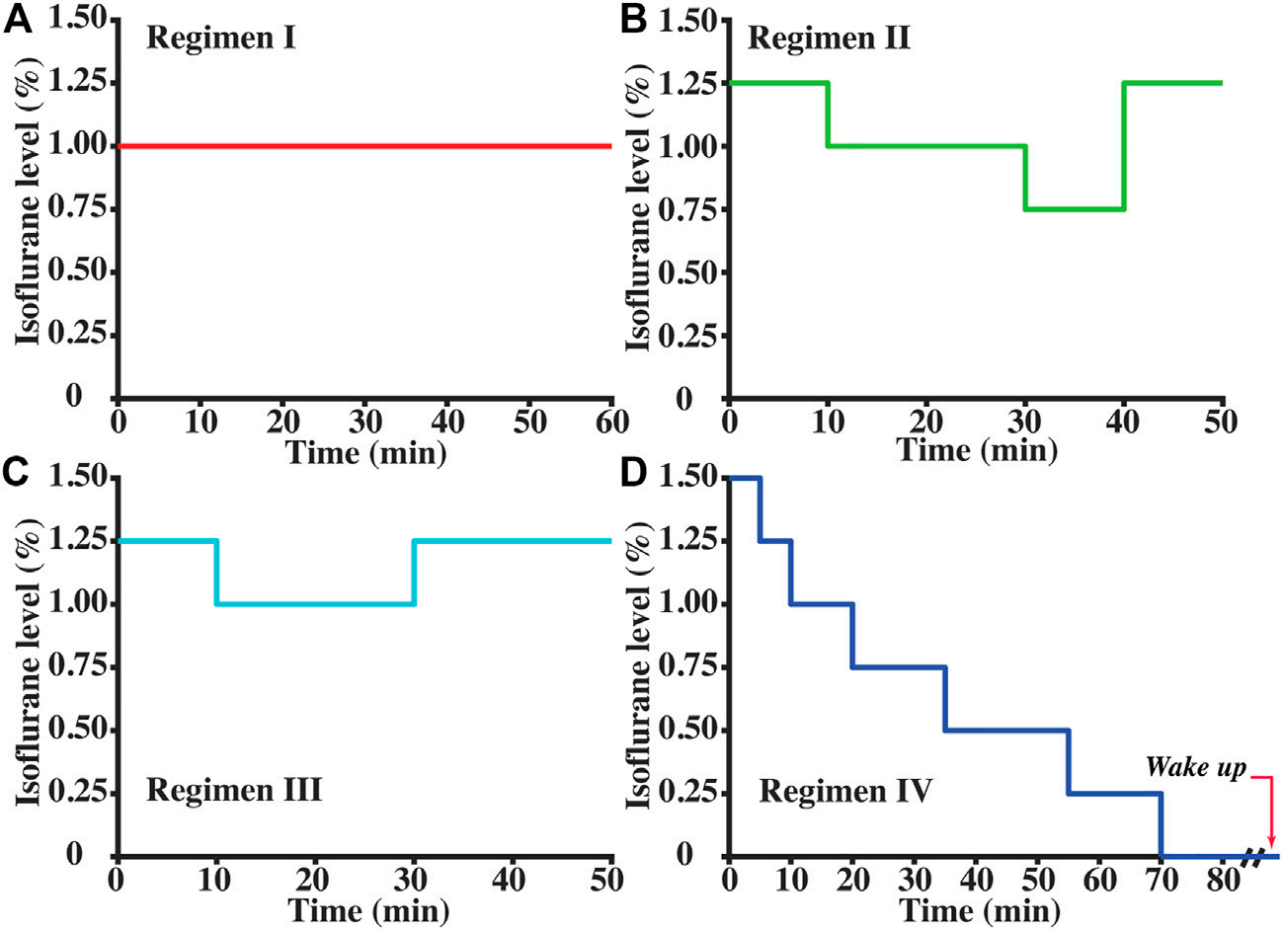
Anesthetic regimens to explore the relationships between CBF and other basic parameters (isoflurane dose, exposure time to anesthetic regimen, respiration rate, and heart rate): **(A)** Regimen I (N = 5); **(B)** Regimen II (N = 4); **(C)** Regimen III (N = 4); and **(D)** Regimen IV (N = 6). Isoflurane was delivered with medical air (21% oxygen) at 0.5 L/min.

Our previous study suggested that the effects of anesthesia on brain physiology not only depend on the dose but also on how long the anesthesia has been applied to the animal (Wei et al., 2022). Therefore, here we systematically studied the time courses of CBF under a variety of dose combinations, which is either used in previous studies or designed to expand the dynamic ranges of the observed physiological parameters, as follows: (a) Regimen I (Figure 1A, N = 5 mice) with a constant 1.00% isoflurane, which is a commonly used regimen in prior studies (Schroeter et al., 2014; Wei et al., 2020); (b) Regimen II (Figure 1B, N = 4) with time-varying isoflurane doses ranging from 0.75% to 1.25%; (c) Regimen III (Figure 1C, N = 4) with time-varying isoflurane doses ranging from 1.00% to 1.25%; (d) Regimen IV (Figure 1D, N = 6) with progressively reducing isoflurane from 1.50% to 0%. Experiments with Regimen IV will result in the mouse waking up toward the end of the session. We, therefore, stopped the CBF scanning when mice exhibited severe motions. Throughout all experimental sessions, anesthesia was delivered with the medical air (21% oxygen and 78% nitrogen) at a constant flow rate of 0.5 L/min.

**FIGURE 2:**
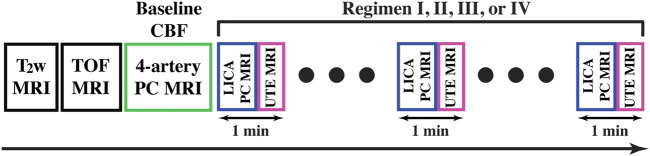
Schematic diagram of the experiments under different anesthetic regimens (I, II, III, and IV). T_2_-weighted (T_2_w) MRI was performed for evaluating brain volume and time-of-flight (TOF) MRI was performed for visualizing arteries. Baseline CBF was measured with 4-artery PC MRI prior to the planned anesthetic regimens. LICA PC and UTE sequences were utilized in an interleaved manner with a temporal resolution of 1 min.

**FIGURE 3:**
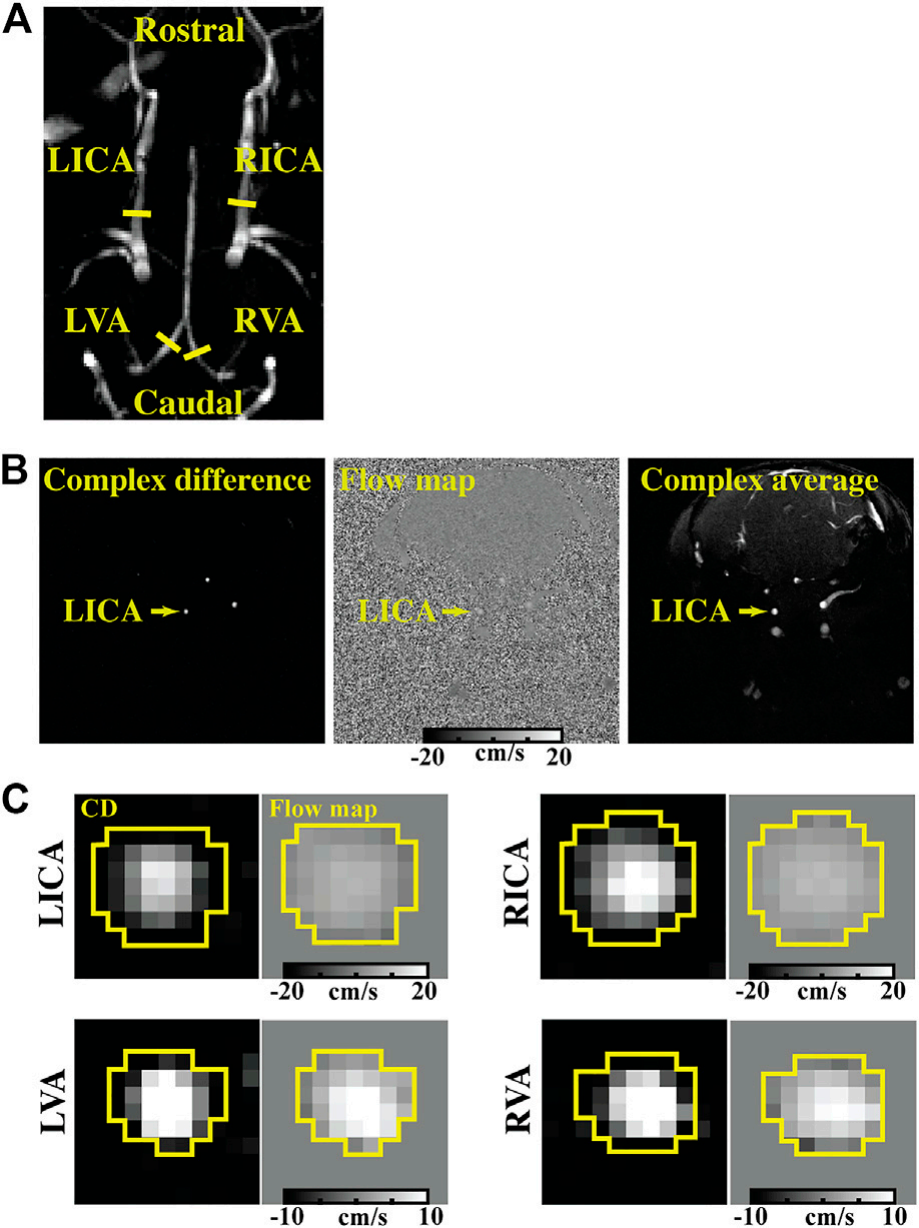
Representative dataset of PC MRI. **(A)** shows the TOF image displaying the slice locations of PC MRI (yellow solid lines perpendicular to the vessel trajectory) focusing on different arteries (LICA, RICA, LVA, and RVA). **(B)** shows the complex difference (CD), flow map, and complex average images of a PC scan focusing on the LICA. **(C)** presents the CD images and flow maps of four feeding arteries. ROIs for quantifying blood flow were delineated on the CD images (yellow boxes) and then applied to the flow maps.

CBF was evaluated with quantitative flow phase-contrast (PC) MRI. The experimental procedure is illustrated in Figure 2. Prior to the PC scans, we performed a coronal TOF angiogram (7 slices, slice thickness = 0.5 mm, no inter-slice gap, TR/TE = 45/2.6 ms, scan duration = 2.0 min) to visualize the feeding arteries (Figure 3A). Next, a sagittal TOF (single slice, tilted to contain the target artery identified from coronal TOF images, thickness = 0.5 mm, TR/TE = 60/2.5 ms, scan duration = 0.4 min) was applied to visualize the in-plane trajectory of each targeted artery. PC MRI was then positioned using both TOFs and performed with the following parameters: TR/TE = 15/3.2 ms, FOV = 15 × 15 mm^2^, matrix size = 300 × 300, slice thickness = 0.5 mm, number of averages = 4, dummy scan = 8, receiver bandwidth = 100 kHz, flip angle = 25°, partial Fourier acquisition factor = 0.7, and scan duration = 0.6 min (Wei et al., 2019). Before starting the planned anesthetic regimens, baseline CBF in each mouse was evaluated with four separate PC scans covering the four major feeding arteries (left/right internal carotid artery, LICA/RICA; left/right vertebral artery, LVA/RVA) of brain (Wei et al., 2019). The total blood flow from LICA, RICA, LVA, and RVA was then normalized by brain weight, which was obtained from a T_2_-weighted fast-spin-echo MRI protocol (TR/TE = 4,000/26 ms, FOV = 15 × 15 mm^2^, matrix size = 128 × 128, slice thickness = 0.5 mm, echo spacing = 5 ms, 35 axial slices, and scan duration = 1.1 min), to gain unit-mass CBF (in a unit of ml/100 g/min) (Wei et al., 2021). For convenience, this baseline CBF was dubbed CBF_0_. After starting the anesthetic regimen (i.e., Regimen I, II, III, or IV), PC MRI was applied to the LICA only in order to obtain a higher temporal resolution (0.6 min as opposed to 2.4 min), and the single-artery CBF value was converted to a unit-mass value by referencing to the *CBF*_0_, i.e., $CBF=\frac{BF_{LICA}}{BF_{LICA,0}}\times CBF_{0}$ , where *BF*_*LICA*_ denoted the dynamic blood flow in LICA measured under different anesthetic regimens and *BF*_*LICA,*0_ denoted the baseline blood flow in LICA of that mouse.

Respiration rate (RR) was monitored with an air cushion, which was connected to a monitoring system (MouseOX), placed under the chest of the mouse while allowing free breathing. Heart rate (HR) was recorded with an MRI method, i.e., an ultrashort TE (UTE) MRI sequence, to avoid needle penetrations into the limbic muscle and thereby minimize the potential physiological perturbation to the mouse (Wei et al., 2020). In the UTE sequence, the center *k*-space was repeatedly recorded at an interval of 8.0 ms to produce a time course of MR signal, the period of which was the R-R interval. The scan duration of a single UTE MRI was 0.4 min. LICA PC and UTE MRI were interleaved (Figure 2) to obtain a simultaneous and dynamic measurements of CBF and HR. RR values were recorded for each pair of PC and UTE scans.

### 2.2 Data processing

All processing was conducted using custom-written MATLAB (MathWorks, Natick, MA) scripts. PC MRI data were processed according to a previous report (Wei et al., 2019). Briefly, the artery of interest was first manually delineated on the complex-difference image, which showed an excellent contrast between the vessel and surrounding tissue (e.g., Figure 3B). The mask was then applied to the velocity map and blood flow (mL/min) through that artery was calculated using the integration of arterial voxels.

The brain volume was estimated using T_2_-weighted images. Briefly, we first manually delineated the brain boundary on a slice-by-slice basis by reference to a mouse brain atlas (https://atlas.brain-map.org/) as reported (Wei et al., 2020). Numbers of voxels inside the masks were summed to yield the total brain volume in mm^3^. The total brain volume was converted into brain mass based on an assumed brain tissue density [1.04 g/mL (Leithner et al., 2010)].

To obtain unit-mass CBF_0_, the total blood flow from PC MRI of the four feeding arteries was divided by brain mass. The CBF_0_ was used to scale the dynamic PC flow data, as described above, assuming that the relative fractions of blood flow across the four arteries remain unchanged during the dynamic data acquisition.

### 2.3 Statistical analyses

The time dependences of CBF under different anesthetic regimens were investigated with linear regression where CBF was the dependent variable and time was the independent variable. To elucidate the relationship between CBF and isoflurane dose, between RR and isoflurane dose, and between HR and isoflurane dose, linear regression was utilized with dose as the independent variable and CBF, RR, or HR as the dependent variable, respectively. To further investigate the dependence of CBF on RR, HR, and dose, a step-wise multi-linear regression analysis was conducted with CBF as the dependent variable and RR, HR, and dose were independent variables. The significant variables were included as predictors for a multi-linear regression model where CBF was the response, i.e., $CBF=\underset{P_{\xi }<0.05}{\sum }C_{i}\xi$ , where C_i_ denoted the coefficient of a predictor, ξ denoted the predictors with significant effect. All data will be used for fitting the coefficients C_i_. The obtained calibration can further be normalized by a reference CBF value to match cases with relative CBF measurements. A *p*-value smaller than 0.05 was considered statistically significant.

## 3 Results

Figure 3 presents the TOF image, complex difference image and velocity map of a representative LICA, RICA, LVA, and RVA PC dataset. It can be seen that the arteries are evident in the complex difference images (Figure 3B), which facilitate the delineation of regions-of-interest (ROIs) (as shown by the yellow circles in Figure 3C) and quantification of CBF.

**FIGURE 4:**
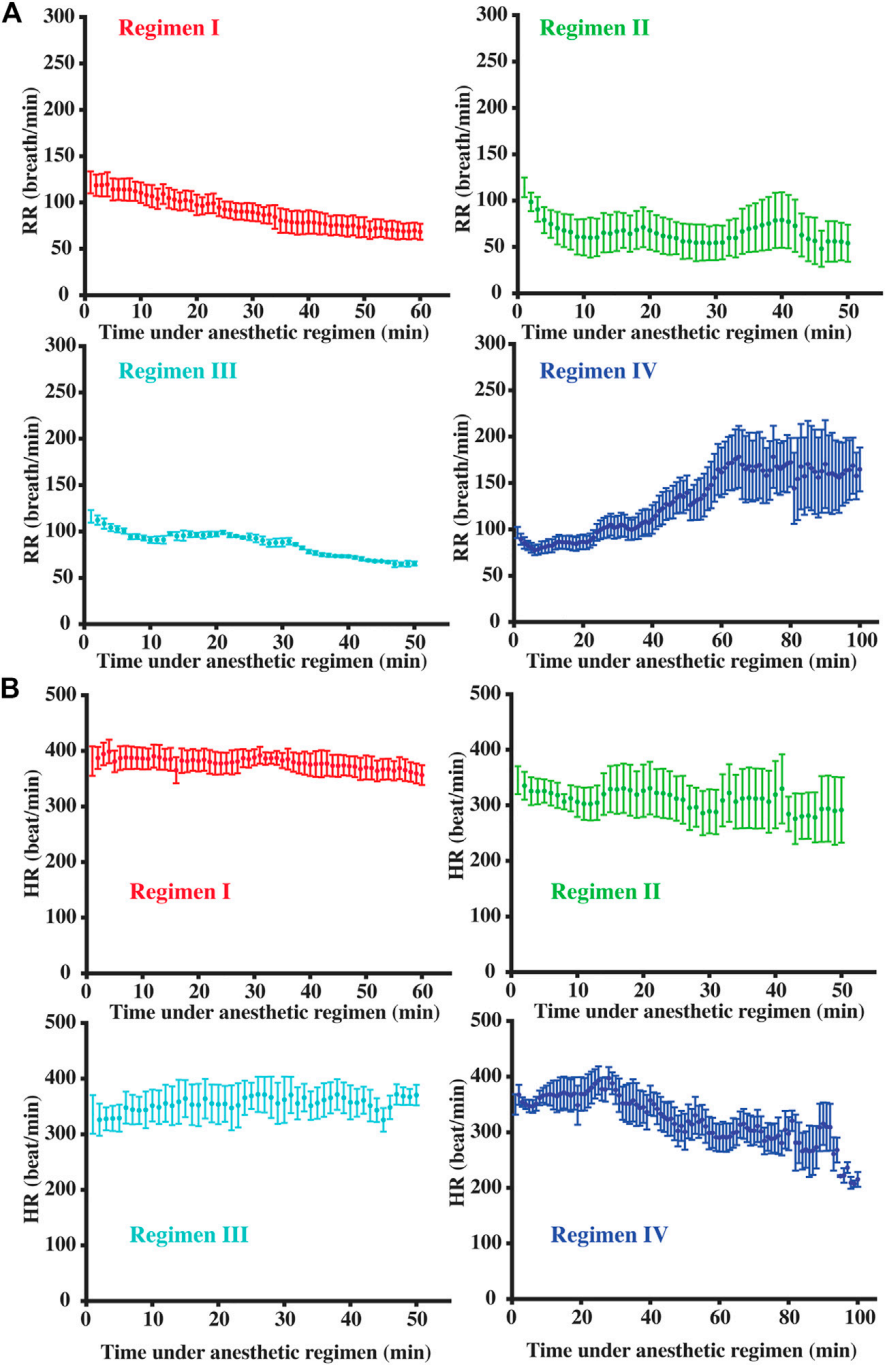
Temporal dynamic respiration rate **(A)** and heart rate **(B)** courses under different anesthetic regimens. Error bar denoted the standard error across mice.

**FIGURE 5:**
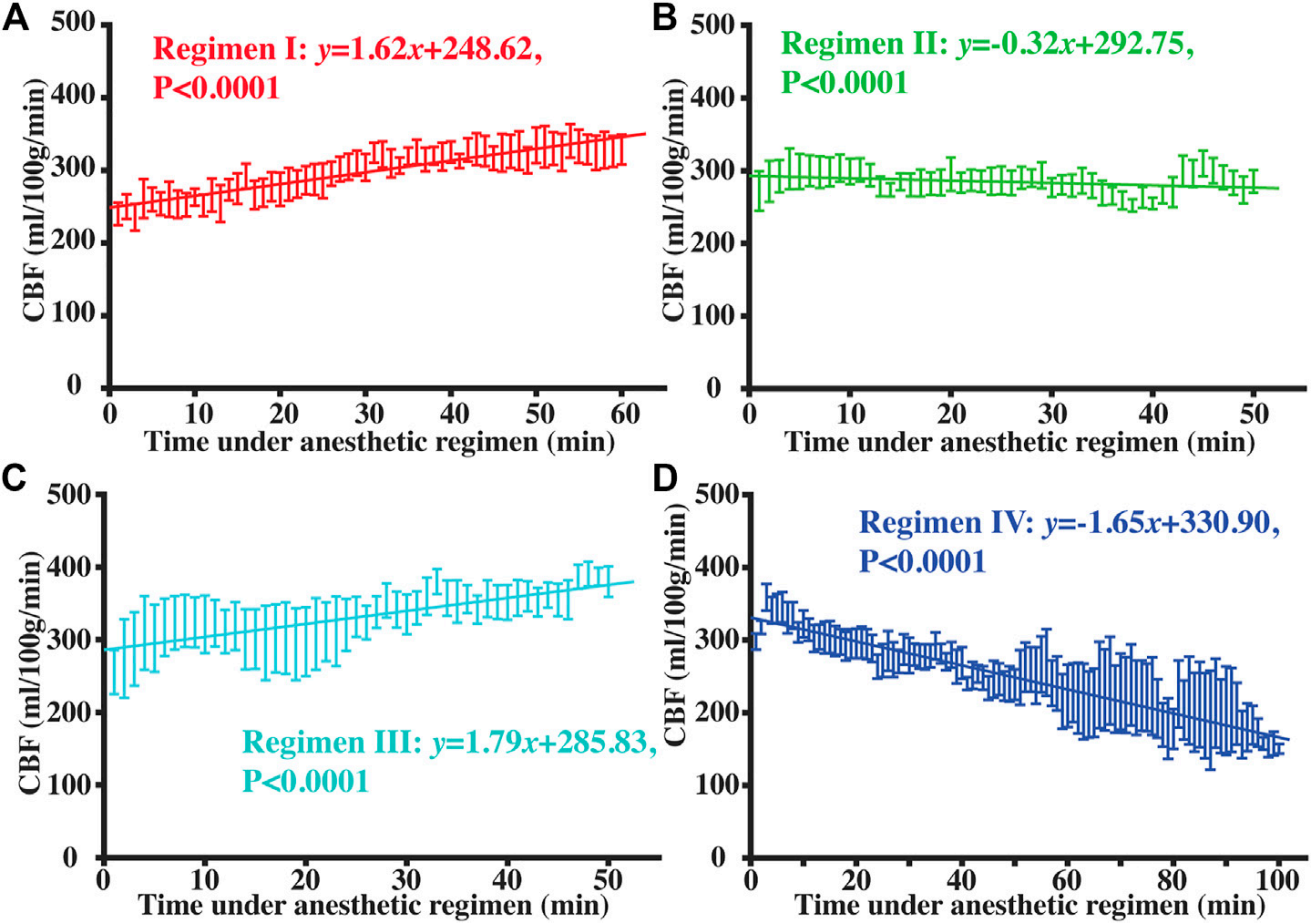
Temporal dynamic curves of CBF under Regimen I **(A)**, II **(B)**, III **(C)**, and IV **(D)**. Error bar showed the standard error of CBF values across mice at each time point, and solid lines represented the fitted equations with linear regression model.

Figure 4 shows RR and HR time-courses during each of the four Regimens. Figure 5 shows the corresponding time-courses for CBF. It can be seen that the patterns of physiological variations are more complex than the anesthetic dose. Even in Regimen I in which the anesthetic dose was held constant, physiological parameters showed a clear time alteration. Therefore, dosage in itself is not sufficient to explain the modulation of CBF by the anesthetic agent.

**FIGURE 6:**
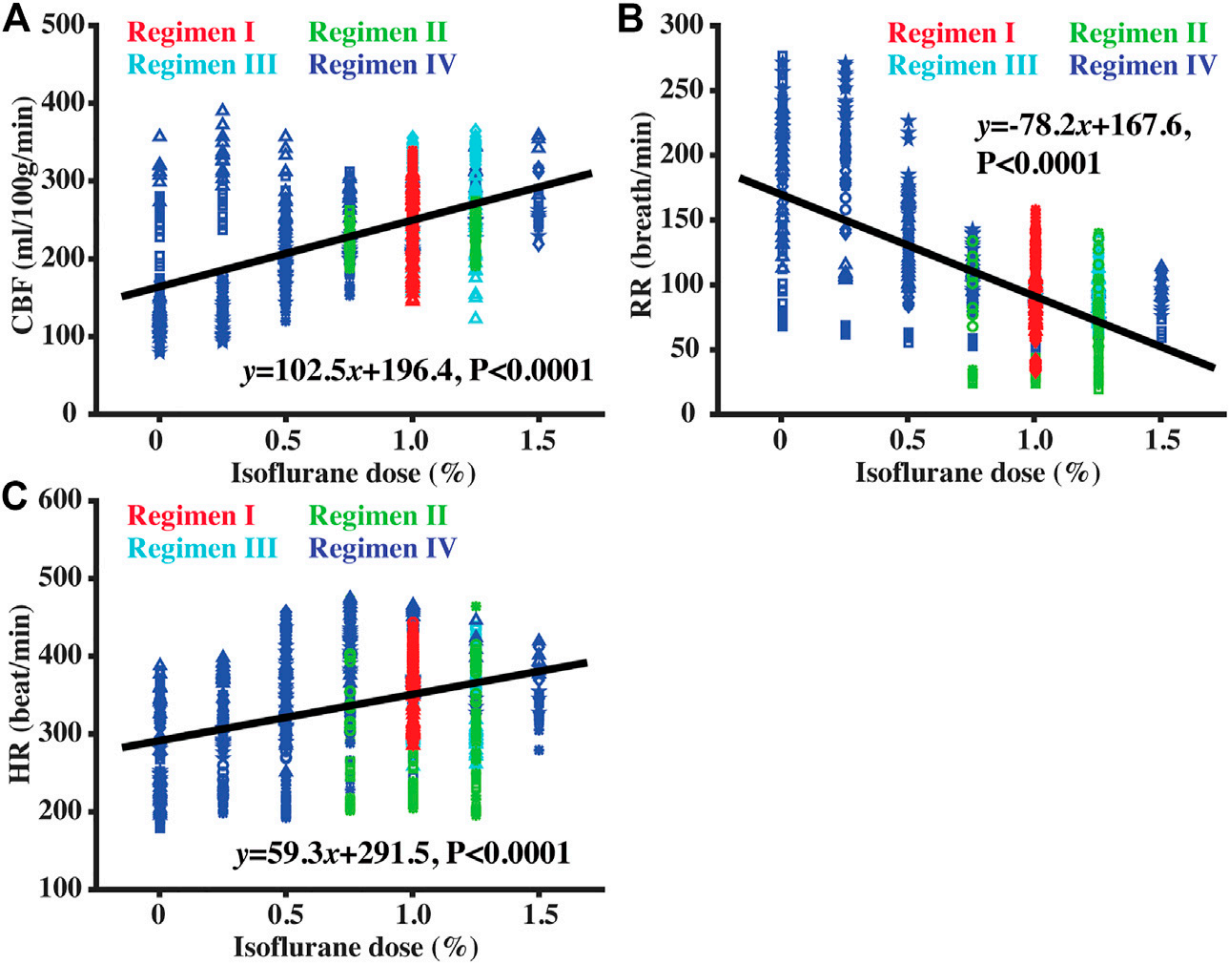
Scatter plots between isoflurane dose and CBF **(A)**, RR **(B)**, and HR **(C)**. Red, green, cyan, and blue symbols corresponded to data collected under Regimens I, II, III, and IV, respectively. Solid lines denoted the fitted equations with linear regression model.

Figure 6 shows the correlations between isoflurane dose and CBF, RR, and HR, when combining data from all four Regimens. CBF was positively correlated with isoflurane dose (Figure 6A, *p* < 0.0001), consistent with the expected vasodilatory effects of isoflurane (Li et al., 2013). However, the coefficient of determination of the correlation (R^2^ = 0.324) was modest. Additionally, there was a significant negative correlation between RR and dose (Figure 6B, *p* < 0.0001) and a significant positive correlation between HR and dose (Figure 6C, *p* < 0.0001).

**FIGURE 7:**
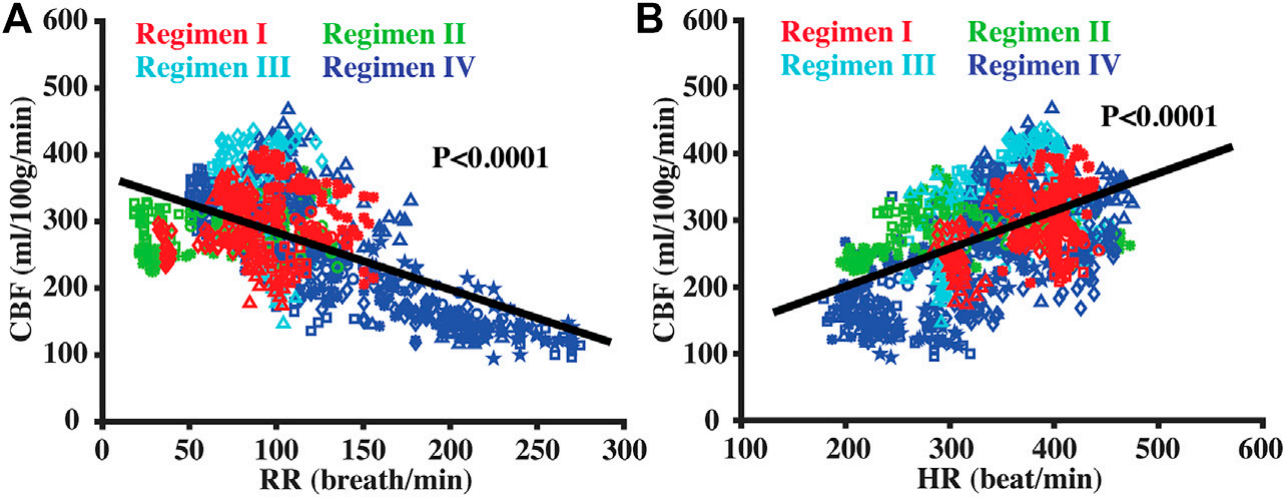
Scatter plots between CBF and RR **(A)** and between CBF and HR **(B)**. Red, green, cyan, and blue symbols corresponded to data collected under Regimens I, II, III, and IV, respectively. Solid lines denoted the fitted equations with linear regression model.

Figure 7 shows the relationships between CBF and RR or HR. There was a significant negative correlation between CBF and RR (Figure 7A, *p* < 0.0001), and a positive correlation between CBF and HR (Figure 7B, *p* < 0.0001). By including both RR and HR into a linear regression model, it was found that both RR and HR can explain a significant amount of variations in CBF data. Note that the above regression model can account for R^2^ = 0.526 of the variances in our entire data, greater than that when using the anesthesia dose (R^2^ = 0.324). If the anesthesia dose was further added to the model, R^2^ increased to 0.544 and the final model became CBF=174.25−0.58RR+0.41HR+32.66Dose , where CBF is in the unit of ml/100 g/min, RR is in the unit of breath/min, HR is in the unit of beat/min, and dose is in %. This model can provide a calibration method to estimate anesthesia-independent CBF for anesthetized mice. When the exposure time was added into the regression model, improvement in R^2^ was not significant (*p* = 0.24).

## 4 Discussion

To the best of our knowledge, the present report is the first MRI study to systematically investigate quantitative measurements of rodent CBF in the context of anesthesia effects. We showed that anesthesia dose in itself cannot fully characterize this effect. Respiration rate and heart rate, which presumably provide a real-time index of the depth of anesthesia, are needed to better explain the CBF variance. An empirical model to correct the anesthesia effect, specifically ${CBF}_{corrected}=CBF+0.58RR-0.41HR-32.66Dose$ , was proposed to provide an anesthesia-corrected CBF estimation. This calibration approach is expected to reduce the physiological variations in CBF data and facilitate CBF comparisons under pathological conditions.

The vasodilative effect of isoflurane on the brain is well-known. It is thought to be due to the modulation of isoflurane on ATP-sensitive K^+^ channels and increased production of endothelium-derived nitric oxide and prostanoids after isoflurane exposure (Moore et al., 1994; Iida et al., 1998). It has also been shown that the anesthesia effect is time-dependent, even under a constant dose (e.g., inhaling gas mixture with 1% isoflurane). This is because the net isoflurane concentration in mice depends on both the clearance and uptake processes. As a result, the physiological state of the animal is not always at a steady state with constant isoflurane concentration. Accordingly, it is expected that CBF change during an anesthesia regimen is a dynamic process. The present study found that RR and HR are important parameters to explain the CBF variations in a typical, hour-long MRI experiment. The reason that RR and HR can explain CBF variance may be: (a) activities of medullary neurons, which mediates breathing and cardiac rate (Salo et al., 2009), were dynamically affected by isoflurane; therefore heart rate and respiration rate are indirect markers for isoflurane concentration; (b) respiration rate is associated with end-tidal CO_2_ level (Takaki et al., 2017), which has a well-known effect on CBF. Note that it is technically challenging to measure end-tidal CO_2_ directly in mice without invasive procedures. Under a general non-invasive setup sampling and detecting gas, the fast respiration rate and low tidal volume in mice will hinder the accurate determination of end-tidal CO_2_ with severe diffusion effect. Mechanical ventilation can improve the measurement accuracy of end-tidal CO_2_, but it is invasive and can increase the risk of lung injuries (Wolthuis et al., 2009). Respiration is the basis of gas exchange in lung. When respiration rate is suppressed under isoflurane, end-tidal CO_2_ level will increase due to insufficient gas exchange, which is supported by the notion of increased end-tidal CO_2_ under breath holding (Bright and Murphy, 2013). As a result, CBF will increase, showing consistency with the dose-dependent vasodilative property of isoflurane. In addition, our findings of a decreasing RR and an increasing HR with increasing isoflurane doses were consistent with previous reports (Picker et al., 2001; Constantinides et al., 2011; Ewald et al., 2011).

CBF is an early biomarker in many brain diseases and has a relatively large effect size. A pathology can alter CBF by up to 80% before structural changes in the brain occur (Fan et al., 2022). Unfortunately, normal variations in CBF also tend to be large, potentially masking out the disease effects. In humans, the variations could be due to differences in breathing patterns, intake of caffeine or medications, or body temperatures. In rodent studies, a major contributor to the CBF variations is anesthesia, which is used in virtually all imaging studies of mice (Muir et al., 2008; Munting et al., 2019; Wei et al., 2019). As a result, a wide range of CBF values have been reported in mice. For example, C57BL/6 mice were reported to have a CBF of ∼200 mL/100 g/min under 1.0% isoflurane (Wei et al., 2020) and ∼280 mL/100 g/min under 1.2% isoflurane (Wei et al., 2023). The present report proposed a model to correct the anesthesia effect on CBF by considering not only dose but also related physiological variables of RR and HR, which are broadly available in animal studies. The proposed model can therefore benefit many mouse studies by providing an anesthesia-corrected CBF estimation.

Along with this goal, we also aimed to provide a reference value of awake-state CBF in mice. As a reminder, in Regimen IV of this study, we gradually reduced the anesthesia dose until the mouse woke up. We were able to acquire some CBF data after the mice woke up but before severe motion occurred, and found a global CBF value of 142.0 ± 8.8 mL/100 g/min. These experimental values are highly consistent with those estimated using the model, which found an awake-state CBF of 148.6 mL/100 g/min when using an averaged awake-state HR and RR of 243 beat/min and 216 breath/min, respectively. Note that these CBF values in the awake state of mice are substantially lower than values reported under anesthetized states (Evans et al., 2020; Wei et al., 2020).

As a technical consideration, PC MRI was used in the current study to measure CBF due to its short scan duration and immunity to arterial transit time influence. However, it should be pointed out that the applicability of the correction model is not limited to the PC MRI, because blood flow is a physiological parameter and should not be affected by the choice of imaging modality. However, it is expected that, due to differences in signal modeling and assumptions used, there may be systematic differences in absolute CBF values across different techniques (e.g., PC MRI versus arterial spin labeling MRI). To extend our model to other quantitative or semi-quantitative (i.e., an arbitrary value of CBF index) CBF techniques, we also derived a correction model for relative CBF. Specifically, we first converted absolute CBF (in ml/100 g/min) to relative CBF by referencing awake-state CBF. After normalizing CBF by the awake-state CBF (i.e., 148.6 mL/100 g/min), the calibration equation becomes CBF=117.26+0.28HR−0.39RR+21.98Dose (unit: %).

We point out that, although the present study focused on correcting the anesthesia effects on CBF measurement, similar correction approaches can be developed for other cerebral physiological parameters such as oxygen extraction fraction (Wei et al., 2018), cerebral metabolic rate of oxygen (CMRO_2_) (Lu et al., 2011), and cerebrovascular reactivity (CVR) (Wei et al., 2022). Furthermore, findings from the present study may also be relevant for clinical studies of patients who are undergoing anesthesia, e.g., during the MR-guided laser interstitial thermal therapy of drug-resistant epilepsy (Lewis et al., 2015). Physiological measurements, e.g., CBF, should be interpreted in the context of the respiration rate and heart rate of these patients.

For a proof-of-principle demonstration of our calibration scheme, we tried to perform the calibration equation ( ${CBF}_{corrected}=CBF+0.58RR-0.41HR-32.66Dose$ ) in a retrospective dataset (N = 10 mice), which recorded CBF twice (gapped by 30 min) by PC MRI with RR recorded. Since the HR information was unavailable, it was estimated based on the HR-RR correlation with a linear regression, i.e., HR=367.91−0.27RR , which changed the calibration equation to be ${CBF}_{corrected}=CBF+0.69RR-32.66Dose$ . A significant improvement (*p* = 0.003) has been noticed by comparing the coefficient-of-variation (CoV) values in original CBF (11.9% ± 2.6%) and in corrected CBF (8.3% ± 2.0%).

This study has a few limitations. First, different anesthetic agents will alter cerebral physiology in different manners, e.g., dexmedetomidine, which is vasoconstrictive to reduce CBF, has an opposite effect in comparison with isoflurane, which is vasodilative to increase CBF (Munting et al., 2019). It is, therefore, not directly feasible to apply the proposed correction scheme to CBF data collected under other anesthesia types. Instead, similar experiments as in the present study can be performed to obtain a specific correction model. Second, despite our best effort, the proposed correction model can still only explain 54% of the total variance in CBF data. There is still approximately half of the variance in the data that cannot be accounted for. Apart from the vasoactive effect, isoflurane has also been reported to decrease metabolism level (Oshima et al., 2003). Therefore, metabolism alterations under isoflurane may partly account for the unexplained variations via the flow-metabolism coupling. Cerebral blood pressure and body temperature may also influence CBF measurements by affecting the cerebral hemodynamics (Croughwell et al., 1992; Smirl et al., 2016). Further work towards these directions is needed to better understand the remaining differences in CBF values within and across animals. Finally, the awake state at the end of Regimen IV where mice recovered from anesthesia could be different from the awake state without any anesthesia history due to the residual effect of isoflurane, e.g., lactate accumulation after long exposure. It is technically challenging but ideal to run experiments with behaviorally adapted animals without anesthetic exposure as reference.

## 5 Conclusion

Variations in CBF values measured in mice can be explained by differences in respiration rate and heart rate, in addition to the anesthesia dose. Therefore, the accuracy of CBF measurement in mice can be improved by using a correction model that accounts for these physiological factors.

## Data Availability

The raw data supporting the conclusion of this article will be made available by the authors, without undue reservation.

## References

[B1] Bright M. G. Murphy K. (2013). Reliable quantification of BOLD fMRI cerebrovascular reactivity despite poor breath-hold performance. Neuroimage 83, 559–568. 10.1016/j.neuroimage.2013.07.007 23845426 PMC3899001

[B2] Constantinides C. Mean R. Janssen B. J. (2011). Effects of isoflurane anesthesia on the cardiovascular function of the C57BL/6 mouse. ILAR J. 52, e21–e31. 21677360 PMC3508701

[B3] Copen W. A. Schaefer P. W. Wu O. (2011). MR perfusion imaging in acute ischemic stroke. Neuroimaging Clin. N. Am. 21, 259–283. 10.1016/j.nic.2011.02.007 21640299 PMC3135980

[B4] Croughwell N. Smith L. R. Quill T. Newman M. Greeley W. Kern F. (1992). The effect of temperature on cerebral metabolism and blood flow in adults during cardiopulmonary bypass. J. Thorac. Cardiovasc Surg. 103, 549–554. 10.1016/s0022-5223(19)34997-9 1545554

[B5] De Visscher G. Verreth W. Blockx H. van Rossem K. Holvoet P. Flameng W. (2006). Application of NIRS in mice: A study comparing the oxygenation of cerebral blood and main tissue oxygenation of mice and rat. Adv. Exp. Med. Biol. 578, 197–202. 10.1007/0-387-29540-2_32 16927693

[B6] Ericsson A. C. Crim M. J. Franklin C. L. (2013). A brief history of animal modeling. Mo Med. 110, 201–205. 23829102 PMC3979591

[B7] Evans P. G. Sokolska M. Alves A. Harrison I. F. Ohene Y. Nahavandi P. (2020). Non-Invasive MRI of blood–cerebrospinal fluid barrier function. Nat. Commun. 11, 2081. 10.1038/s41467-020-16002-4 32350278 PMC7190825

[B8] Ewald A. J. Werb Z. Egeblad M. (2011). Monitoring of vital signs for long-term survival of mice under anesthesia. Cold Spring Harb. Protoc. 2011, 5563. 10.1101/pdb.prot5563 PMC3531964 21285263

[B9] Fan J. L. Brassard P. Rickards C. A. Nogueira R. C. Nasr N. McBryde F. D. (2022). Integrative cerebral blood flow regulation in ischemic stroke. J. Cerebr Blood F. Met. 42, 387–403. 10.1177/0271678X211032029 PMC8985438 34259070

[B10] Fukuda M. Vazquez A. L. Zong X. Kim S. G. (2013). Effects of the α₂-adrenergic receptor agonist dexmedetomidine on neural, vascular and BOLD fMRI responses in the somatosensory cortex. Eur. J. Neurosci. 37, 80–95. 10.1111/ejn.12024 23106361 PMC3538949

[B11] Hedna V. S. Ansari S. Shahjouei S. Cai P. Y. Ahmad A. S. Mocco J. (2015). Validity of laser Doppler flowmetry in predicting outcome in murine intraluminal middle cerebral artery occlusion stroke. J. Vasc. Interv. Neurol. 8, 74–82. 26301036 PMC4535598

[B12] Hirschler L. Debacker C. S. Voiron J. Kohler S. Warnking J. M. Barbier E. L. (2018). Interpulse phase corrections for unbalanced pseudo-continuous arterial spin labeling at high magnetic field. Magn. Reson Med. 79, 1314–1324. 10.1002/mrm.26767 28585234

[B13] Iida H. Ohata H. Iida M. Watanabe Y. Dohi S. (1998). Isoflurane and sevoflurane induce vasodilation of cerebral vessels via ATP-sensitive K+ channel activation. Anesthesiology 89, 954–960. 10.1097/00000542-199810000-00020 9778013

[B14] Kawabata K. Tachibana H. Sugita M. (1991). Cerebral blood flow and dementia in Parkinson's disease. J. Geriatr. Psychiatry Neurol. 4, 194–203. 10.1177/089198879100400404 1789907

[B15] Kisler K. Nelson A. R. Montagne A. Zlokovic B. V. (2017). Cerebral blood flow regulation and neurovascular dysfunction in Alzheimer disease. Nat. Rev. Neurosci. 18, 419–434. 10.1038/nrn.2017.48 28515434 PMC5759779

[B16] Lassen N. A. (1959). Cerebral blood flow and oxygen consumption in man. Physiol. Rev. 39, 183–238. 10.1152/physrev.1959.39.2.183 13645234

[B17] Leithner C. Muller S. Fuchtemeier M. Lindauer U. Dirnagl U. Royl G. (2010). Determination of the brain-blood partition coefficient for water in mice using MRI. J. Cerebr Blood F. Met. 30, 1821–1824. 10.1038/jcbfm.2010.160 PMC3023928 20842161

[B18] Lewis E. C. Weil A. G. Duchowny M. Bhatia S. Ragheb J. Miller I. (2015). MR-guided laser interstitial thermal therapy for pediatric drug-resistant lesional epilepsy. Epilepsia 56, 1590–1598. 10.1111/epi.13106 26249524

[B19] Li C. X. Patel S. Auerbach E. J. Zhang X. (2013). Dose-dependent effect of isoflurane on regional cerebral blood flow in anesthetized macaque monkeys. Neurosci. Lett. 541, 58–62. 10.1016/j.neulet.2013.02.007 23428509 PMC4349366

[B20] Liu H. Zhang C. Xu J. Jin J. Cheng L. Miao X. (2021). Huntingtin silencing delays onset and slows progression of huntington’s disease: A biomarker study. Brain 144, 3101–3113. 10.1093/brain/awab190 34043007 PMC8634120

[B21] Lotz J. Meier C. Leppert A. Galanski M. (2002). Cardiovascular flow measurement with phase-contrast MR imaging: Basic facts and implementation. RadioGraphics 22, 651–671. 10.1148/radiographics.22.3.g02ma11651 12006694

[B22] Lu H. Xu F. Rodrigue K. M. Kennedy K. M. Cheng Y. Flicker B. (2011). Alterations in cerebral metabolic rate and blood supply across the adult lifespan. Cereb. Cortex 21, 1426–1434. 10.1093/cercor/bhq224 21051551 PMC3097991

[B23] Mathew R. J. Wilson W. H. (1991). Substance abuse and cerebral blood flow. Am. J. Psychiatry 148, 292–305. 10.1176/ajp.148.3.292 1992832

[B24] Moore L. E. Kirsch J. R. Helfaer M. A. Tobin J. R. McPherson R. W. Traystman R. J. (1994). Nitric oxide and prostanoids contribute to isoflurane-induced cerebral hyperemia in pigs. Anesthesiology 80, 1328–1337. 10.1097/00000542-199406000-00021 7516628

[B25] Muir E. R. Shen Q. Duong T. Q. (2008). Cerebral blood flow MRI in mice using the cardiac-spin-labeling technique. Magn. Reson Med. 60, 744–748. 10.1002/mrm.21721 18727091 PMC2581653

[B26] Munting L. P. Derieppe M. P. P. Suidgeest E. Denis de Senneville B. Wells J. A. van der Weerd L. (2019). Influence of different isoflurane anesthesia protocols on murine cerebral hemodynamics measured with pseudo-continuous arterial spin labeling. NMR Biomed. 32, e4105. 10.1002/nbm.4105 31172591 PMC6772066

[B27] Oshima T. Karasawa F. Okazaki Y. Wada H. Satoh T. (2003). Effects of sevoflurane on cerebral blood flow and cerebral metabolic rate of oxygen in human beings: A comparison with isoflurane. Eur. J. Anaesthesiol. 20, 543–547. 10.1017/s0265021503000863 12884987

[B28] Peterson E. C. Wang Z. Britz G. (2011). Regulation of cerebral blood flow. Int. J. Vasc. Med. 2011, 823525. 10.1155/2011/823525 21808738 PMC3144666

[B29] Picker O. Scheeren T. W. Arndt J. O. (2001). Inhalation anaesthetics increase heart rate by decreasing cardiac vagal activity in dogs. Br. J. Anaesth. 87, 748–754. 10.1093/bja/87.5.748 11878527

[B30] Salo L. M. Nalivaiko E. Anderson C. R. McAllen R. M. (2009). Control of cardiac rate, contractility, and atrioventricular conduction by medullary raphe neurons in anesthetized rats. Am. J. Physiol. Heart Circ. Physiol. 296, H318–H324. 10.1152/ajpheart.00951.2008 19074673 PMC2643892

[B31] Schroeter A. Schlegel F. Seuwen A. Grandjean J. Rudin M. (2014). Specificity of stimulus-evoked fMRI responses in the mouse: The influence of systemic physiological changes associated with innocuous stimulation under four different anesthetics. Neuroimage 94, 372–384. 10.1016/j.neuroimage.2014.01.046 24495809

[B32] Slupe A. M. Kirsch J. R. (2018). Effects of anesthesia on cerebral blood flow, metabolism, and neuroprotection. J. Cerebr Blood F. Met. 38, 2192–2208. 10.1177/0271678X18789273 PMC6282215 30009645

[B33] Smirl J. D. Hoffman K. Tzeng Y. C. Hansen A. Ainslie P. N. (2016). Relationship between blood pressure and cerebral blood flow during supine cycling: Influence of aging. J. Appl. Physiol. 120, 552–563. 10.1152/japplphysiol.00667.2015 26586907 PMC4773644

[B34] Takaki S. Mizutani K. Fukuchi M. Yoshida T. Idei M. Matsuda Y. (2017). Deep breathing improves end-tidal carbon dioxide monitoring of an oxygen nasal cannula-based capnometry device in subjects extubated after abdominal surgery. Respir. Care 62, 86–91. 10.4187/respcare.04634 27899530

[B35] Wei Z. Chen L. Hou X. van Zijl P. C. M. Xu J. Lu H. (2020). Age-related alterations in brain perfusion, venous oxygenation, and oxygen metabolic rate of mice: A 17-month longitudinal MRI study. Front. Neurol. 11, 559. 10.3389/fneur.2020.00559 32595596 PMC7304368

[B36] Wei Z. Chen L. Lin Z. Jiang D. Xu J. Liu P. (2019). Optimization of phase-contrast MRI for the estimation of global cerebral blood flow of mice at 11.7T. Magn. Reson Med. 81, 2566–2575. 10.1002/mrm.27592 30393888 PMC6372341

[B37] Wei Z. Li Y. Hou X. Han Z. Xu J. McMahon M. T. (2022). Quantitative cerebrovascular reactivity MRI in mice using acetazolamide challenge. Magn. Reson Med. 88, 2233–2241. 10.1002/mrm.29353 35713368 PMC9574885

[B38] Wei Z. Liu H. Lin Z. Yao M. Li R. Liu C. (2023). Non-contrast assessment of blood-brain barrier permeability to water in mice: An arterial spin labeling study at cerebral veins. Neuroimage 268, 119870. 10.1016/j.neuroimage.2023.119870 36640948 PMC9908858

[B39] Wei Z. Xu J. Chen L. Hirschler L. Barbier E. L. Li T. (2021). Brain metabolism in tau and amyloid mouse models of Alzheimer's disease: An MRI study. NMR Biomed. 34, e4568. 10.1002/nbm.4568 34050996 PMC9574887

[B40] Wei Z. Xu J. Liu P. Chen L. Li W. van Zijl P. C. M. (2018). Quantitative assessment of cerebral venous blood T2 in mouse at 11.7T: Implementation, optimization, and age effect. Magn. Reson Med. 80, 521–528. 10.1002/mrm.27046 29271045 PMC5910286

[B41] Wolthuis E. K. Vlaar A. P. Choi G. Roelofs J. J. Juffermans N. P. Schultz M. J. (2009). Mechanical ventilation using non-injurious ventilation settings causes lung injury in the absence of pre-existing lung injury in healthy mice. Crit. Care 13, R1. 10.1186/cc7688 19152704 PMC2688111

[B42] Yeom K. W. Mitchell L. A. Lober R. M. Barnes P. D. Vogel H. Fisher P. G. (2014). Arterial spin-labeled perfusion of pediatric brain tumors. Am. J. Neuroradiol. 35, 395–401. 10.3174/ajnr.A3670 23907239 PMC7965744

